# Prognostic value of APOC1 in patients with acute-on-chronic liver failure

**DOI:** 10.3389/fimmu.2026.1881095

**Published:** 2026-10-02

**Authors:** Hongyan Peng, Geng Zhang, Lianyu He, Bo Wang, Li Qiang, Yunjian Sheng, Wen Chen, Honglian Du, Xiaoli Tian, Qian Zhang

**Affiliations:** Department of Infectious Diseases, The Affiliated Hospital of Southwest Medical University, Luzhou, Sichuan, China

**Keywords:** ACLF1, apolipoprotein C15, DIA2, ELISA3, prognostic biomarker4, saikosaponin D6

## Abstract

**Background:**

Acute-on-chronic liver failure (ACLF) is associated with high short-term mortality. Valid prognostic biomarkers are required to refine risk stratification and improve clinical management. This study aimed to identify potential prognostic biomarkers for predicting clinical outcomes in ACLF patients.

**Methods:**

Seventy-four ACLF patients admitted to the Affiliated Hospital of Southwest Medical University between January 2021 and April 2022 were enrolled for retrospective analysis of baseline characteristics, clinical parameters and clinical outcomes. Forty patients were randomly selected for data-independent acquisition mass spectrometry (DIA-MS)-based proteomic profiling and differential protein analysis, and categorized into survival (n = 20) and non-survival (n = 20) groups according to 90-day outcomes. Bioinformatics analyses were performed to characterize candidate targets. Apolipoprotein C1 (APOC1) levels were validated by enzyme-linked immunosorbent assay (ELISA). Receiver operating characteristic (ROC) curve analysis was used to evaluate its prognostic performance and compare it with a newly established model, conventional biomarkers and existing scoring systems. Single-cell RNA sequencing was applied to determine the cellular source and distribution of APOC1, and molecular docking was performed to screen potential APOC1-targeted therapeutic agents.

**Results:**

Proteomic analysis identified 41 differentially expressed proteins (DEPs). Protein–protein interaction network analysis revealed that APOC1 occupied a core hub position in the network. Functional enrichment analysis indicated that DEPs were mainly involved in lipid metabolism, lipoprotein clearance and cholesterol efflux. ELISA confirmed significantly higher plasma APOC1 levels in the survival group, suggesting a protective role. Comparative ROC analysis demonstrated favorable prognostic performance of APOC1. Incorporation of APOC1 into traditional scoring systems moderately improved predictive efficacy. Single-cell RNA sequencing further revealed that APOC1 was predominantly expressed in hepatic macrophages. Molecular docking showed strong binding affinities of APOC1 with saikosaponin D and resveratrol, with binding energies of −7.8 and −6.7 kcal/mol, respectively. Elevated plasma APOC1 was positively correlated with survival in ACLF patients.

**Conclusion:**

Plasma APOC1 serves as a novel prognostic biomarker for ACLF. Integrating APOC1 into conventional scoring systems yields complementary prognostic value. Hepatic macrophages represent the primary cellular source of APOC1, and saikosaponin D and resveratrol are promising candidate agents targeting APOC1.

## Introduction

Worldwide, approximately 2 million people die from liver disease each year, accounting for nearly 4% of all-cause mortality, with the majority of these deaths attributable to chronic liver disease and its related complications. Owing to its large population base, China bears an especially heavy burden of liver disease, with an estimated 340–480 million individuals affected by chronic liver diseases (1, 2). ACLF is a complex clinical syndrome that occurs in patients with underlying chronic liver disease and is triggered by various precipitating factors. It is characterized by rapidly progressive jaundice and coagulopathy and may be further complicated by ascites, hepatic encephalopathy, infection, renal insufficiency, and multiple organ failure. Because of its abrupt onset, rapid progression, and high short-term mortality, ACLF has become one of the leading causes of death among patients with chronic liver disease (3, 4).

The pathophysiology of ACLF is highly complex and is primarily characterized by acute decompensation superimposed on chronic liver injury. In this setting, liver failure, systemic inflammation, and multiorgan dysfunction interact and mutually amplify one another, ultimately forming a vicious cycle (3–6). On the one hand, chronic liver disease compromises the functional reserve of hepatocytes, and exposure to precipitating insults may induce extensive hepatocyte necrosis, leading to a rapid decline in hepatic synthetic, metabolic, and detoxification functions. On the other hand, damage-associated molecular patterns (DAMPs) released from necrotic hepatocytes activate the innate immune system, triggering a cytokine storm and systemic inflammatory response syndrome (SIRS). This process not only exacerbates hepatic injury but also affects extrahepatic organs, including the circulatory, respiratory, and renal systems, thereby accelerating disease progression (4–8). Recent clinical studies have shown that even with intensive individualized interventions, such as comprehensive medical therapy and artificial liver support, the 90-day transplant-free mortality of patients with ACLF remains unacceptably high, and prognosis worsens markedly with increasing numbers of failed organs. Therefore, the early identification of molecular biomarkers associated with prognosis is of considerable clinical importance for improving risk stratification, guiding treatment decisions, and ultimately enhancing short-term survival outcomes in patients with ACLF (3, 4).

Currently, the prognostic assessment of ACLF in clinical practice mainly relies on models based on clinical manifestations and routine laboratory parameters, including the Model for End-Stage Liver Disease (MELD), MELD-Na, and the Chinese Group on the Study of Severe Hepatitis B-ACLF (COSSH-ACLF) score (9–12). Although these scoring systems are useful for evaluating disease severity and predicting clinical outcomes to some extent, they are still largely dependent on conventional clinical variables and fail to fully capture the dynamic molecular alterations that occur during disease progression. Consequently, their performance remains limited in terms of precise risk stratification and mechanism-based prognostic evaluation (3, 11, 12).

The swift advancement of high-throughput omics technologies has made genomics, transcriptomics, proteomics, and metabolomics potent tools for discovering biomarkers (13–15). Among these approaches, proteomics enables the systematic characterization of protein expression profiles and their dynamic changes under pathological conditions. By comparing protein expression patterns between healthy controls and patients, or between patient subgroups with different clinical outcomes, proteomics offers a valuable strategy for identifying biomarkers with potential diagnostic and prognostic significance (13, 14). Compared with genomic analyses, proteins more directly reflect the functional state of the organism and the ongoing pathological processes, thereby conferring unique advantages in prognostic studies of ACLF (16).

Against this background, the present study aimed to identify and validate prognostic biomarkers associated with ACLF. First, plasma samples from patients with ACLF were analyzed using DIA mass spectrometry to systematically characterize prognosis-related alterations in protein expression. Candidate targets were subsequently screened through bioinformatics analyses, and the key candidate biomarker APOC1 was further validated by ELISA.

APOC1 is the smallest member of the apolipoprotein C family, with a molecular weight of approximately 6.6 kDa. It is a small secreted protein predominantly synthesized by hepatocytes and plays an important role in lipid metabolism (17). Previous studies have shown that APOC1 regulates triglyceride and very low-density lipoprotein (VLDL) metabolism by inhibiting lipoprotein lipase (LPL) and hepatic lipase (HL), while also participating in the modulation of inflammatory responses (17). More recently, accumulating evidence has suggested that APOC1 is involved in maintaining hepatic synthetic function and lipid homeostasis. In patients with acute liver failure, APOC1 levels are markedly reduced and are closely associated with impaired hepatocellular synthetic function. In a single-cell sequencing study of HBV-related acute-on-chronic liver failure (HBV-ACLF), APOC1 was identified as a key gene associated with TREM2+ macrophage differentiation and lipid metabolic reprogramming, and its altered expression may reflect disturbances in the hepatic immune microenvironment and metabolic homeostasis (18). Notably, in an experimental model of alcoholic hepatitis, downregulation of the hepatocyte nuclear factor 4α (HNF4α)-dependent gene APOC1 was closely associated with hepatocellular dysfunction, suggesting that APOC1 may act as an important protective factor in maintaining hepatocyte homeostasis (19).

Based on these observations, the present study was designed to: (1) determine the expression level of APOC1 in the serum of patients with ACLF and assess its correlation with clinical parameters; (2) investigate the association between APOC1 expression and disease severity as well as short-term prognosis in ACLF; (3) evaluate the predictive performance of APOC1 as an independent prognostic factor and compare it with conventional prognostic scoring systems. Through these analyses, we sought to provide a novel and precise molecular biomarker for prognostic assessment in ACLF and to offer an experimental basis for the development of individualized therapeutic strategies. (**Figure 1**).

**Figure 1:**
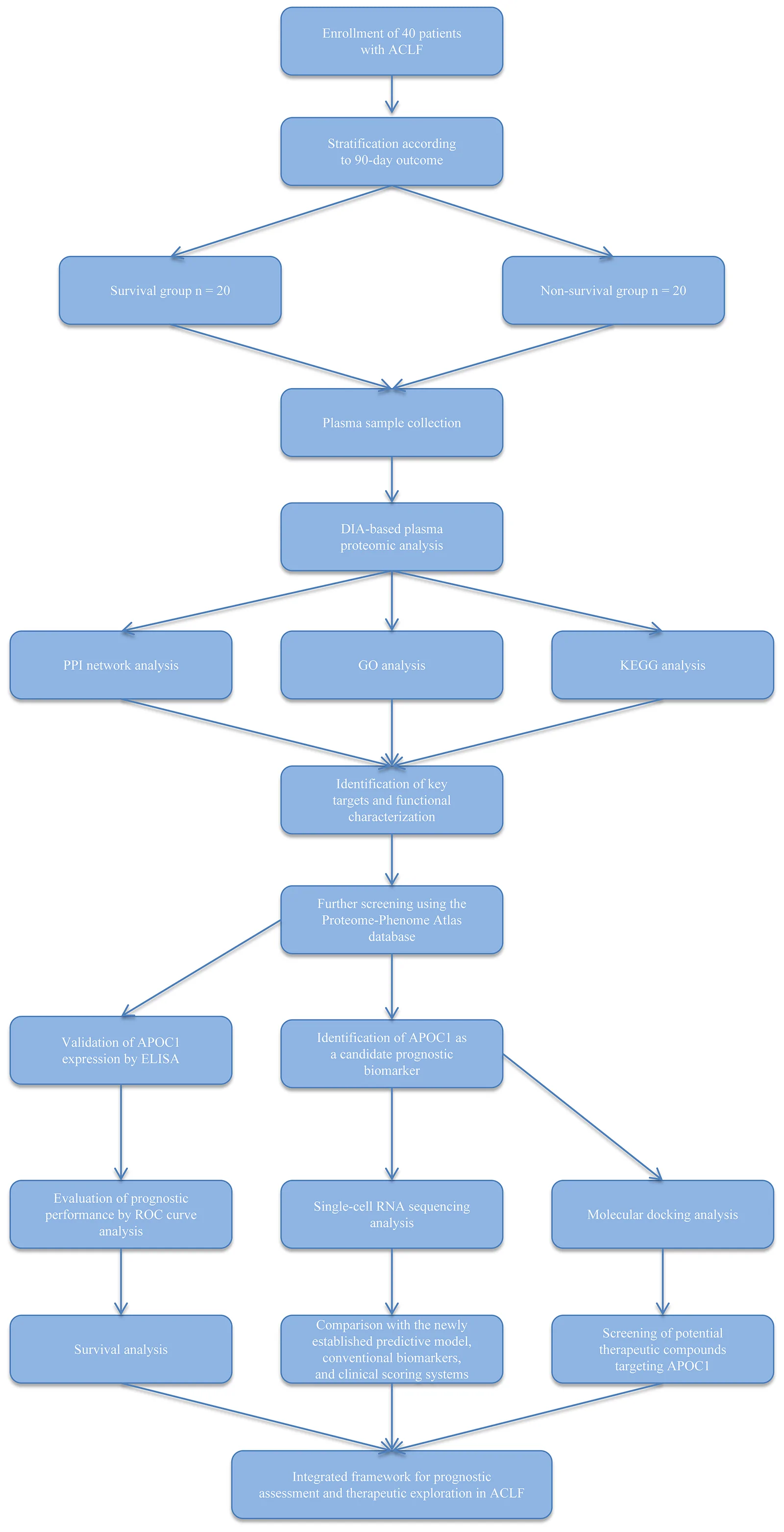
Flowchart of research methods.

## Materials and methods

### Blood sample and clinical data collection in ACLF patients

Peripheral blood samples were collected from patients with HBV-ACLF who were admitted to the Department of Infectious Gastroenterology, the Affiliated Hospital of Southwest Medical University, between January 2021 and April 2022. The diagnosis of HBV-ACLF was based on the criteria of the Chinese Guidelines for the Diagnosis and Treatment of Liver Failure (2018 edition), defined as serum total bilirubin (TBil) ≥12 mg/dL (205.2 μmol/L) or a daily increase of ≥1 mg/dL (17.1 μmol/L), together with an international normalized ratio (INR) ≥1.5 or prothrombin activity (PTA) ≤40%. All patients were followed up for 90 days or until death. The exclusion criteria were as follows: (1) unwillingness to participate in the study; and (2) the presence of other viral infections, including human immunodeficiency virus (HIV), hepatitis A virus (HAV), hepatitis C virus (HCV), or hepatitis E virus (HEV), or hepatocellular carcinoma. Blood samples were obtained within 24h after admission. Plasma was collected after centrifugation of peripheral blood and stored at -80 °C until further analysis. The Ethics Committee of the Affiliated Hospital of Southwest Medical University (No. KY2021014) reviewed this study. Consent and signatures were obtained from all patients or their families. The clinical trial is registered under the number ChiCTR2100042896 at the Chinese Clinical Trial Registry (www.chictr.org.cn).

Clinical data were collected, including sex, age, MELD score, MELD-Na score, COSSH-II score, total white blood cell count, neutrophil count, platelet count, hemoglobin, urea, creatinine, alanine aminotransferase (ALT), aspartate aminotransferase (AST), serum total bilirubin, direct bilirubin, albumin, INR, prothrombin activity, retinol-binding protein, alpha-fetoprotein, procalcitonin, quantitative hepatitis B virus serological markers, and HBV DNA levels. Statistical analyses were performed using R software (version 4.2.1). Normally distributed continuous variables were indicated by the mean ± standard deviation, whereas non-normally distributed variables were expressed as the median (interquartile range). Comparisons between two groups with normally distributed data were made using the independent-samples t test, and one-way ANOVA was used for comparisons among multiple groups. The Mann-Whitney U test was used for comparing two groups with non-normally distributed data, while the Kruskal-Wallis H test was applied for comparisons among multiple groups. Categorical variables were expressed as frequencies and percentages and were compared using the chi-square test or Fisher’s exact test, as appropriate. A two-sided P < 0.05 was considered statistically significant.

### Proteomic analysis

The clinical cohort comprised 74 patients with acute-on-chronic liver failure (ACLF), including 47 survivors and 27 non-survivors. Forty patients were randomly selected from this cohort for data-independent acquisition (DIA)-based quantitative proteomic analysis, comprising 20 survivors and 20 non-survivors. Proteomic analysis was performed by BGI (Shenzhen, China) according to its standardized experimental and analytical workflow. Briefly, a spectral library was first generated from the experimental samples using data-dependent acquisition (DDA). The library contained all detectable, non-redundant, high-quality peptide tandem mass spectrometry (MS/MS) spectra, together with fragment-ion intensity and retention-time information, and peptide identifications with a false discovery rate (FDR) of ≤1% were retained. Protein extracts were quantified using the Bradford assay with a 0.2 μg/μL bovine serum albumin calibration curve, and absorbance was measured at 595 nm; protein concentrations were calculated from the calibration curve and the corresponding dilution factors. For quality assessment, 10 μg of protein from each sample was heated at 95 °C for 5 min, centrifuged at 25,000 × g for 5 min, and separated on a 12% SDS-polyacrylamide gel at 80 V for 30 min followed by 120 V for 120 min, after which the gel was rapidly stained, destained, and scanned. For enzymatic digestion, approximately 100 μg of protein per sample, with the amount adjusted when required by the specific project, was loaded onto a 10-kDa molecular-weight-cutoff centrifugal filter unit and washed three times with 0.5 M triethylammonium bicarbonate (TEAB) by centrifugation at 12,000 × g and 20 °C for 20 min. Trypsin was then added at a trypsin-to-protein mass ratio of 1:20, and digestion was performed at 37 °C for 4 h. Peptides were collected by centrifugation at 12,000 × g and 20 °C for 15 min, and the remaining peptides were recovered by adding 100 μL of 0.5 M TEAB and repeating the centrifugation; the combined peptide solutions were subsequently lyophilized. The dried peptides were reconstituted in mobile phase A, consisting of 2% acetonitrile (ACN) and 0.1% formic acid (FA), centrifuged at 20,000 × g for 10 min, and separated using a Vanquish Neo liquid chromatography system equipped with an EASY-Spray™ column (150 μm × 15 cm) at flow rates of 0.8–2.5 μL/min. Mobile phase B consisted of 80% ACN and 0.1% FA, and the gradient was programmed as follows: 4%–5% B from 0 to 0.5 min, with the flow rate decreasing from 2.5 to 2.0 μL/min; 5%–8.5% B from 0.5 to 0.9 min, with the flow rate decreasing from 2.0 to 0.8 μL/min; 8.5%–25% B from 0.9 to 14.2 min; 25%–35% B from 14.2 to 21.1 min; 35%–55% B from 21.1 to 21.5 min, with the flow rate increasing from 0.8 to 2.0 μL/min; and 55%–99% B from 21.5 to 21.9 min, with the flow rate increasing from 2.0 to 2.5 μL/min, followed by maintenance at 99% B from 21.9 to 22.7 min. The liquid chromatography system was directly coupled to an Astral mass spectrometer, and the peptides were ionized using a nano-electrospray ionization source and analyzed in DIA mode. The ion-source voltage was set to 1.8 kV. Full MS scans were acquired over an m/z range of 380–980 at a resolution of 240,000 and a maximum injection time of 5 ms. The precursor range of m/z 380–980 was evenly divided into 300 consecutive isolation windows for sequential fragmentation and signal acquisition. Precursor ions were fragmented by higher-energy collisional dissociation with a normalized collision energy of 25; the maximum injection time for MS/MS acquisition was 3 ms, fragment ions were detected using the Astral mass analyzer, and the automatic gain control targets for both MS1 and MS2 were set to 500% (20, 21).

### Differential expression analysis

Data preprocessing was performed using iDEP0.96, and the expression data were log2-transformed (22). Differentially expressed proteins between the survival and non-survival groups of patients with acute-on-chronic liver failure were identified based on the criteria of |FC| > 1.0 and false discovery rate (FDR) < 0.05. Sample distribution and homogeneity were evaluated using boxplots generated with the ggplot2 package.

### GO enrichment and PPI analysis

To further explore the functional roles of the differentially expressed proteins in ACLF, functional enrichment analyses were performed to preliminarily identify the biological processes and signaling pathways in which these proteins may be involved. Gene Ontology (GO) analysis was conducted in three categories: biological process (BP), molecular function (MF), and cellular component (CC), to characterize the biological significance and functional features of the candidate proteins. Kyoto Encyclopedia of Genes and Genomes (KEGG) pathway analysis was further performed to identify their involvement in metabolic and signaling pathways and to screen potential therapeutic targets. To investigate the interactions among the identified differentially expressed proteins, a protein-protein interaction (PPI) network analysis was performed. The PPI network was constructed using the STRING (https://cn.string-db.org/) database to identify hub proteins among the candidate targets. Proteins located closer to the center of the network and connected to more nodes were considered more likely to be core targets. The analysis was restricted to Homo sapiens, with a minimum interaction score of 0.4, and disconnected nodes were removed. The remaining proteins were regarded as potential targets associated with the prognosis of ACLF.

### Proteome-phenome atlas analysis

To further clarify the expression characteristics, clinical correlations, and prognostic value of APOC1 in patients with ACLF, we searched APOC1 in the Proteome-Phenome Atlas database. The Proteome-Phenome Atlas (https://proteome-phenome-atlas.com/) is a public plasma protein-phenotype association repository based on 53,000 UK Biobank adults, covering 2,920 plasma proteins, nearly 1,000 health-related phenotypes, and over 1,000 disease endpoints. It provides high-quality, reusable evidence for large-scale proteomic and disease mechanism research, using Cox proportional hazards regression for incident diseases and logistic regression for prevalent diseases to establish quantitative protein-disease associations (23). Via artificial intelligence and big data, it integrates multi-omics data to explore protein-disease associations, causal evidence, and prognostic values, supporting precision medicine in biomarkers, pathogenesis, and therapeutic targets. In this study, we searched liver failure-related endpoints in the database, extracted statistically significant differential plasma proteins and key data (effect sizes, standard errors, P-values), and imported them into Xiantao Academic (https://www.xiantaozi.com/) for forest plot visualization to show associations between key plasma proteins and liver failure occurrence/progression. This analysis provided reliable omics-based evidence for early warning, risk stratification, mechanistic exploration, and prognostic assessment of liver failure.

### ELISA verification

To further verify the clinical relevance of the potential biomarker, blood samples from volunteers were detected using human APOC1 ELISA kits strictly following the manufacturer’s instructions. Appropriate statistical methods (stats and car packages) were applied based on data distribution characteristics; no statistical analysis was performed if the data did not meet the requirements. Data were visualized with the ggplot2 package.

### Survival analysis

A total of 74 ACLF patients enrolled in this study were strictly divided into the survival group and non-survival group according to clinical outcomes at the follow-up endpoint. The survival group (n=47) was defined as patients who survived without death events during follow-up, while the non-survival group (n=27) included patients who died of ACLF-related complications or disease progression.

Survival analysis was performed to systematically analyze the survival status of the two groups. Specifically, the survival package was used for proportional hazards assumption testing and survival regression fitting. Results were visualized using the survminer and ggplot2 packages to intuitively show the survival time distribution and cumulative survival rate trend of the two groups. Meanwhile, the Log-rank test was used to compare the survival time difference between the two groups, with corresponding test statistics and P values calculated to determine whether there was a statistical difference in survival prognosis between the two groups, laying a statistical foundation for subsequent analysis of factors affecting patient survival prognosis and verification of the clinical significance of the critical state threshold.

### Logistic regression analysis

This study included patients with ACLF who met the diagnostic criteria of the Chinese Guidelines for the Diagnosis and Treatment of Liver Failure (2018 edition). Clinical data were preprocessed, including the imputation of missing values, correction of outliers, and variable encoding. ACLF-related outcomes were designated as the dependent variable, whereas demographic characteristics and laboratory parameters were included as independent variables. Logistic regression analysis was then performed in two stages. First, univariable analyses were conducted, and variables with P < 0.1 were entered into the multivariable analysis. Subsequently, a stepwise procedure was used to construct the multivariable logistic regression model. Model calibration was assessed using the Hosmer-Lemeshow goodness-of-fit test, and discriminative performance was evaluated by the C statistic. In addition, variance inflation factors (VIFs) were calculated to detect multicollinearity among independent variables, thereby ensuring the robustness and reliability of the model.

### ROC curve analysis

ROC curve analysis was performed to evaluate the clinical utility of the newly developed predictive model and the novel biomarker APOC1 for prognostic assessment in patients with ACLF. The predictive performance of the new model and APOC1 was compared with that of conventional laboratory parameters, including total bilirubin and the international normalized ratio (INR), as well as established clinical scoring systems, including the Model for MELD score, MELD-Na score, and COSSH-ACLF II score. The area under the ROC curve (AUC) was calculated to assess discriminative ability, with values closer to 1.0 indicating higher prognostic accuracy.

Comparative analyses were conducted to examine whether the novel biomarker and the new model demonstrated improved prognostic performance relative to traditional assessment tools, and to identify indicators with potential clinical applicability characterized by high sensitivity and specificity. The optimal cut-off value for each indicator was determined based on the maximum Youden index (sensitivity + specificity − 1), corresponding to the point on the ROC curve at which the sum of the false-positive and false-negative rates was minimized. This threshold was used to define the point at which ACLF progressed to a critical state, thereby providing a quantitative basis for the early identification of high-risk patients, assessment of disease severity, and prognostic evaluation in clinical practice.

### Single-cell RNA sequencing

Single-cell RNA-sequencing data were obtained from the GSE136103 dataset in the Gene Expression Omnibus database. The dataset included six participants, comprising three healthy controls and three patients with liver cirrhosis. Multiple liver tissue specimens were collected from each participant. One healthy control and one patient with cirrhosis each contributed three tissue specimens, whereas the remaining two healthy controls and two patients with cirrhosis each contributed two specimens, resulting in a total of 14 liver tissue specimens for sequencing (24). To analyze the core gene expression patterns within distinct cell subpopulations using single-cell transcriptomic data, the following steps were performed: First, batch effects were mitigated using the Harmony algorithm. Next, optimal cell clustering was performed with the SNN clustering method. UMAP dimensionality reduction was then applied to visualize the cellular community structure. Differentially expressed genes were identified with the presto algorithm, focusing on genes with logFC > 0 and min. pct > 0.25 in each cell population. Cell type annotations were assigned using the SingleR database (25). As the dataset did not include samples from patients with acute-on-chronic liver failure (ACLF), it was used solely to explore the cell-type-specific expression pattern of APOC1 in healthy and cirrhotic liver tissues. The results were considered supplementary evidence describing APOC1 expression in the chronic liver injury microenvironment and were not interpreted as direct evidence of its cellular expression pattern in ACLF.

### Virtual screening and molecular docking

A combined virtual screening and molecular docking strategy was used to explore the biological role of APOC1 in ACLF and to assess its potential as a therapeutic target. APOC1 was queried in the Integrated Traditional Chinese Medicine (ITCM) database(http://itcm.biotcm.net/profile.html)and the High-throughput Experiment- and Reference-guided database of Traditional Chinese Medicine 2.0 (HERB 2.0) (http://47.92.70.12/FAQ/)to identify candidate monomer compounds predicted to upregulate APOC1 expression. The top 15 ranked compounds were selected for subsequent analysis. Among these, monomer components present in hepatoprotective compound formulas were further screened. Compounds with weak binding affinity or unfavorable docking energies were excluded based on the docking results. The two-dimensional structures of candidate ligands were obtained from PubChem (https://pubchem.ncbi.nlm.nih.gov/) and converted into three-dimensional conformations using ChemOffice, followed by export in mol2 format. The crystal structure of APOC1 was retrieved from the RCSB Protein Data Bank (http://www.rcsb.org/), and a high-resolution structure was selected as the docking receptor. Protein preprocessing, including removal of water molecules and phosphate ions, was performed using PyMOL 2.6. Receptor and ligand preparation, including hydrogen addition, assignment of ligand torsions, and definition of the docking grid box, was carried out using AutoDock 1.5.6. Molecular docking was then performed using AutoDock Vina, and the optimal binding conformation was determined according to the docking score. The docking results were visualized using Discovery Studio 2019 and PyMOL 2.6 to generate two-dimensional interaction maps and three-dimensional structural views.

## Results

### Clinical information

A total of 74 patients with ACLF were enrolled, including 47 survivors (63.5%) and 27 non-survivors (36.5%). There was no significant difference in gender composition between the two groups (P = 0.312). Non-survivors were significantly older than survivors (50.63 ± 9.18 vs. 44.45 ± 9.40 years, P = 0.008). No significant intergroup differences were observed in lymphocyte count, monocyte count, leukocyte count, neutrophil count, hemoglobin, creatinine, potassium, sodium, chloride, alpha-fetoprotein, procalcitonin, NMLR, MLR, dNLR, NLR, SIRI, or SII (all P>0.05). Survivors had significantly higher platelet count (125 (1) vs. 103 [65.5, 123.5] ×10^9^/L, P = 0.008) and albumin level (32.74 ± 4.27 vs. 29.83 ± 4.52 g/L, P = 0.007), while total bilirubin was significantly lower (297.46 ± 111.09 vs. 387.97 ± 161.01 μmol/L, P = 0.013). No significant differences were found in ALT, AST, or DBIL (all P>0.05). Non-survivors exhibited markedly worse coagulation function, with higher INR (2.85 [2.09, 3.315] vs. 1.76 [1.595, 2.09], P<0.001) and lower PTA (24 [22, 36] vs. 42 [35, 49] %, P<0.001). NPR was significantly higher in non-survivors (0.050 [0.039, 0.078] vs. 0.038 [0.028, 0.047], P = 0.005), whereas PLR showed no significant difference (P = 0.056). Furthermore, non-survivors had significantly higher MELD, MELD-NA, and COSSH-II scores (all P<0.001 or P = 0.001), indicating more severe liver dysfunction and poorer prognosis. The detailed clinical data are presented in **Table 1**.

**Table 1:** Clinically relevant information of the sample.

Characteristics	Survivor(n=47)	Non-survivor(n=27)	P value
Gender, n (%)			0.312
male	30 (40.5%)	14 (18.9%)	
female	17 (23%)	13 (17.6%)	
Age(years)	44.447 ± 9.3966	50.63 ± 9.1825	0.008
lymphocytes (10^9^/L)	1.21 (0.88, 1.7)	1.16 (0.715, 1.785)	0.578
Monocytes (10^9^/L)	0.56 (0.44, 0.775)	0.61 (0.48, 0.805)	0.432
Leukocytes (10^9^/L)	6.78 (5.98, 8.425)	6.89 (5.87, 8.8)	0.937
Neutrophils (10^9^/L)	4.78 (3.925, 6.22)	4.64 (3.27, 6.63)	0.884
HGB(g/L)	133.23 ± 16.263	125.37 ± 22.469	0.087
PLT (10^9^/L)	125 (94.5, 163.5)	103 (65.5, 123.5)	0.008
ALT(U/L)	612.7 (240.05, 1278.5)	532.8 (145.5, 1075.8)	0.320
AST(U/L)	424.1 (180, 1077.2)	293.5 (150.1, 726.6)	0.503
TBIL (μmol/L)	297.46 ± 111.09	387.97 ± 161.01	0.013
DBIL (μmol/L)	210.32 ± 82.203	248.3 ± 126.52	0.170
ALB(g/L)	32.743 ± 4.2688	29.826 ± 4.5179	0.007
CREA(μmol/L)	68.5 (59.4, 79.65)	70.3 (53.85, 91.9)	0.818
INR	1.76 (1.595, 2.09)	2.85 (2.09, 3.315)	< 0.001
PTA (%)	42 (35, 49)	24 (22, 36)	< 0.001
K (mmol/L)	3.8783 ± 0.53726	3.7674 ± 0.53755	0.396
Na (mmol/L)	136.6 (134.05, 138.85)	136.4 (133.6, 139.5)	0.724
Cl (mmol/L)	102.4 (99.6, 105.45)	104.1 (98.75, 107.45)	0.497
AFP (ng/ml)	65.96 (15.85, 143.28)	31.48 (8.22, 93.42)	0.329
PCT (ng/ml)	0.791 (0.5275, 1.4365)	1.02 (0.7045, 2.44)	0.102
NMLR	4.3802 (2.9512, 5.9125)	5.2229 (2.965, 7.7584)	0.396
NPR	0.038087 (0.027689, 0.046518)	0.050325 (0.039315, 0.078133)	0.005
MLR	0.47321 (0.3645, 0.61725)	0.53509 (0.36854, 0.8165)	0.284
PLR	98.462 (79.186, 132.3)	84.699 (52.426, 118.63)	0.056
dNLR	0.86544 ± 0.048629	0.87048 ± 0.042045	0.654
NLR	4.0083 (2.6494, 5.2861)	4.6051 (2.6819, 7.0519)	0.415
SIRI	2.2667 (1.3605, 3.5814)	2.5814 (1.4334, 4.8599)	0.448
SII	462.11 (314.56, 635.98)	438.49 (219.62, 668.98)	0.278
MELD	27.9 (20.95, 33.45)	37.3 (27.9, 40.5)	< 0.001
MELD-NA	29.115 ± 7.3041	35.678 ± 8.9118	0.001
COSSH-II	7.1 (6.4, 7.55)	8.1 (7.45, 8.55)	< 0.001

### Differential protein screening

A total of 40 patients with ACLF were included in this study and stratified according to their 90-day outcomes into a survival group (n = 20) and a non-survival group (n = 20). DIA-based mass spectrometry was performed, and differential expression analysis of the DIA data was conducted to identify potential key prognostic targets. Compared with the non-survival group, 30 proteins were upregulated and 11 proteins were downregulated in the survival group. Among these proteins, APOC1 was upregulated in the survival group, suggesting a potentially protective role (**Figure 2**).

**Figure 2:**
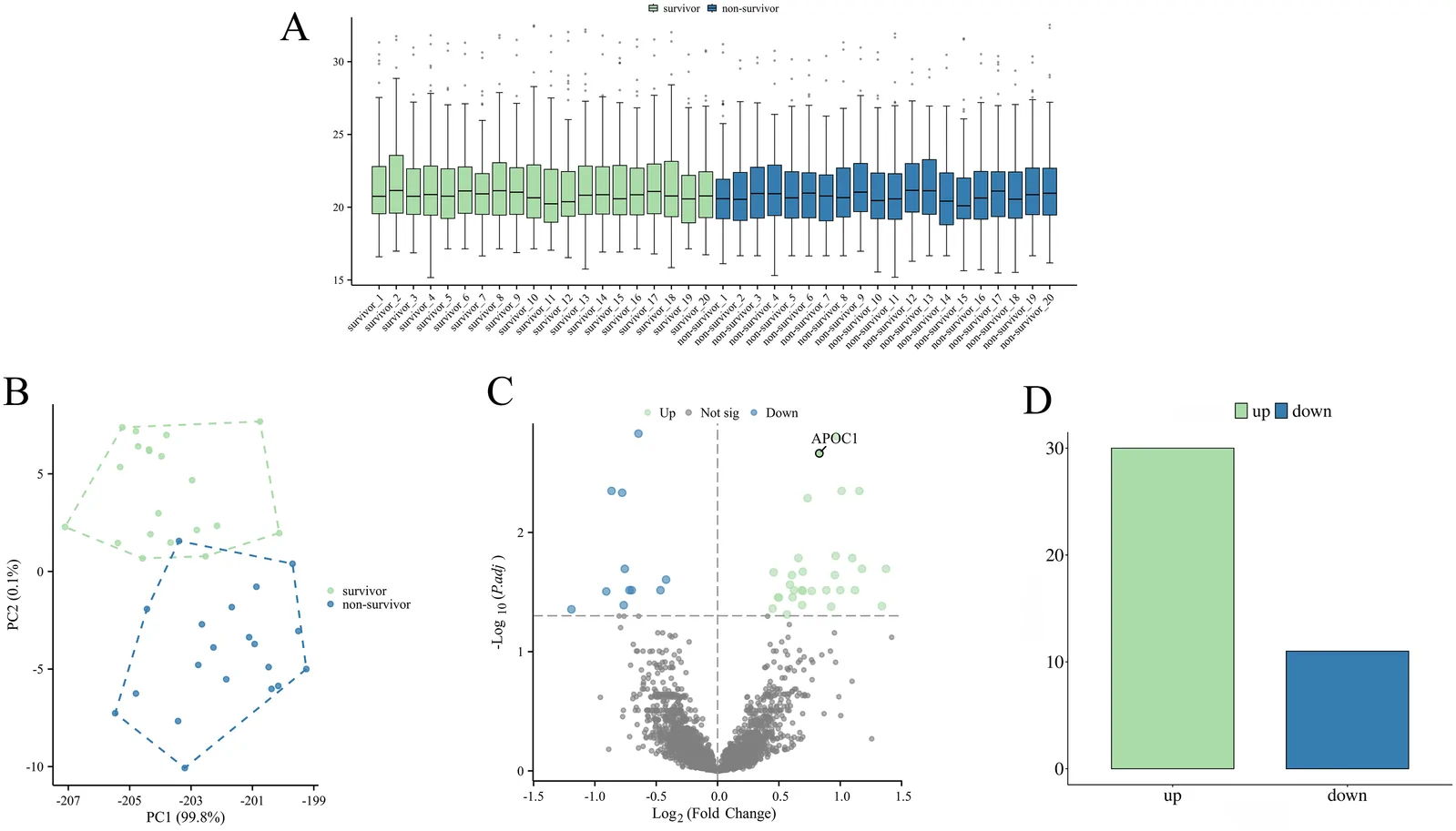
**(A, B)** Log2-normalized DIA proteomic data showed good overall homogeneity and comparability across all samples. **(C)** Volcano plot of differentially expressed proteins. The x-axis indicates logFC and the y-axis indicates −log10(P value). Green and blue dots represent upregulated and downregulated proteins, respectively. In total, 41 differentially expressed proteins were identified, including 30 upregulated and 11 downregulated proteins in the survival group. APOC1 was significantly upregulated in the survival group. **(D)** Bar plot of the differentially expressed proteins.

### Protein-protein interaction network analysis

Differentially expressed proteins were imported into the STRING database to construct a protein-protein interaction (PPI) network. The results showed that most differentially expressed proteins were clustered within the core region of the network and exhibited close interactions with one another. Network topology analysis further indicated that APOC1 occupied a key nodal position within the core module, suggesting that it may play an important role in ACLF progression, although its precise biological function remains to be further elucidated experimentally (**Figure 3**).

**Figure 3:**
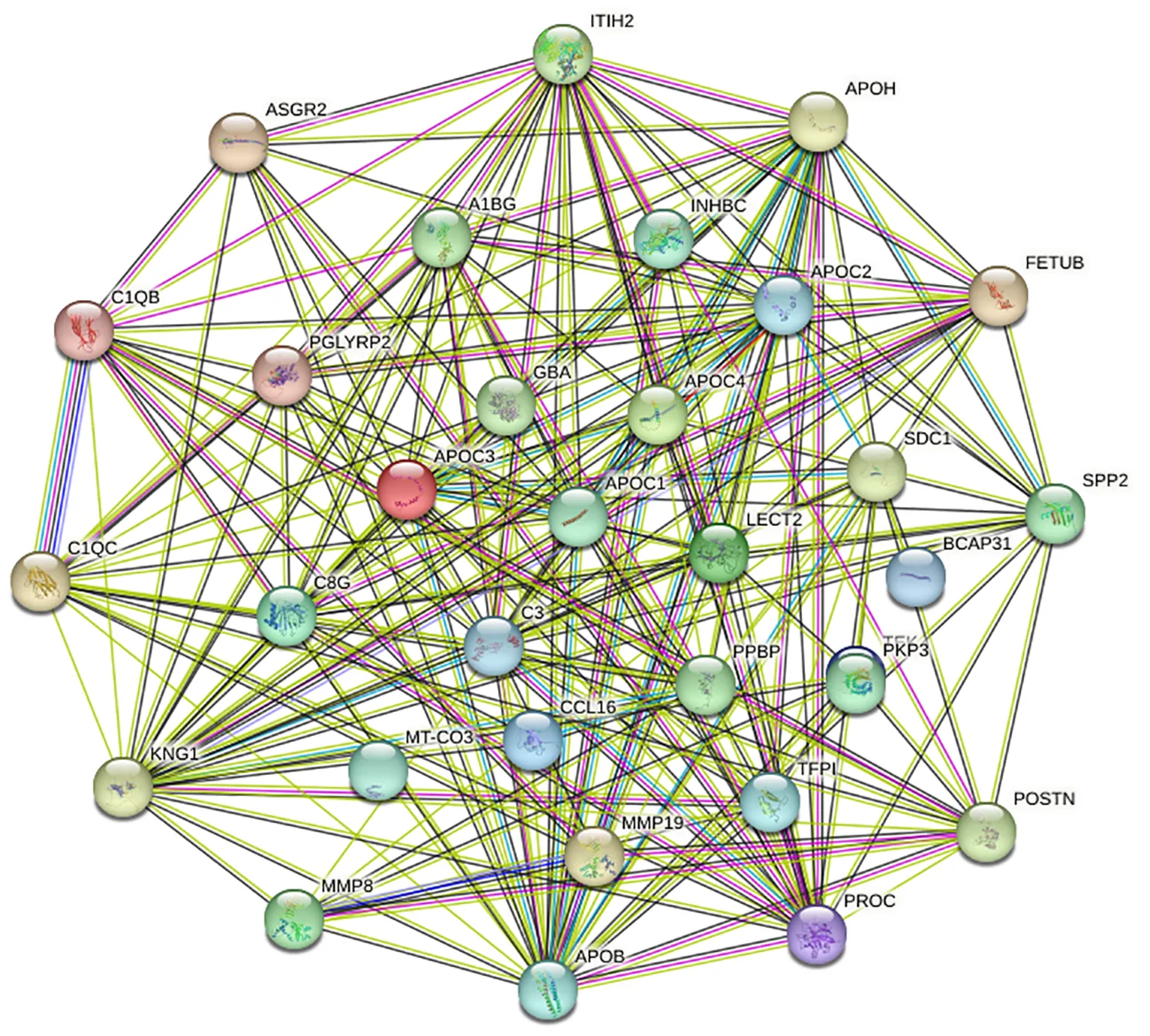
Protein–protein interaction (PPI) network analysis of differentially expressed proteins. In the PPI network, APOC1 was identified as a hub protein located at the core of the network, indicating its potential functional importance and suggesting that it may represent a key target for further research.

### GO and KEGG enrichment analyses

The differentially expressed proteins were significantly enriched in lipid metabolism-related Gene Ontology (GO) biological processes, mainly including plasma lipoprotein particle clearance and remodeling, chylomicron remnant clearance, and cholesterol efflux. In terms of cellular components, these proteins were primarily enriched in VLDL particles, HDL particles, vesicle lumen, and endoplasmic reticulum lumen. At the molecular function level, enzyme inhibitory activity was markedly enriched, with endopeptidase inhibitor activity being particularly prominent. KEGG pathway analysis showed that the differentially expressed proteins were mainly enriched in the cholesterol metabolism pathway. These findings suggest that the identified key proteins may influence disease progression and clinical outcomes in patients with ACLF through the regulation of lipid metabolic processes. Among them, APOC1 is a small plasma protein predominantly synthesized by the liver and widely involved in inflammatory, immune, and other pathophysiological processes. Its biological functions are mainly mediated through the regulation of key enzymes and receptors involved in lipoprotein metabolism (**Figure 4**).

**Figure 4:**
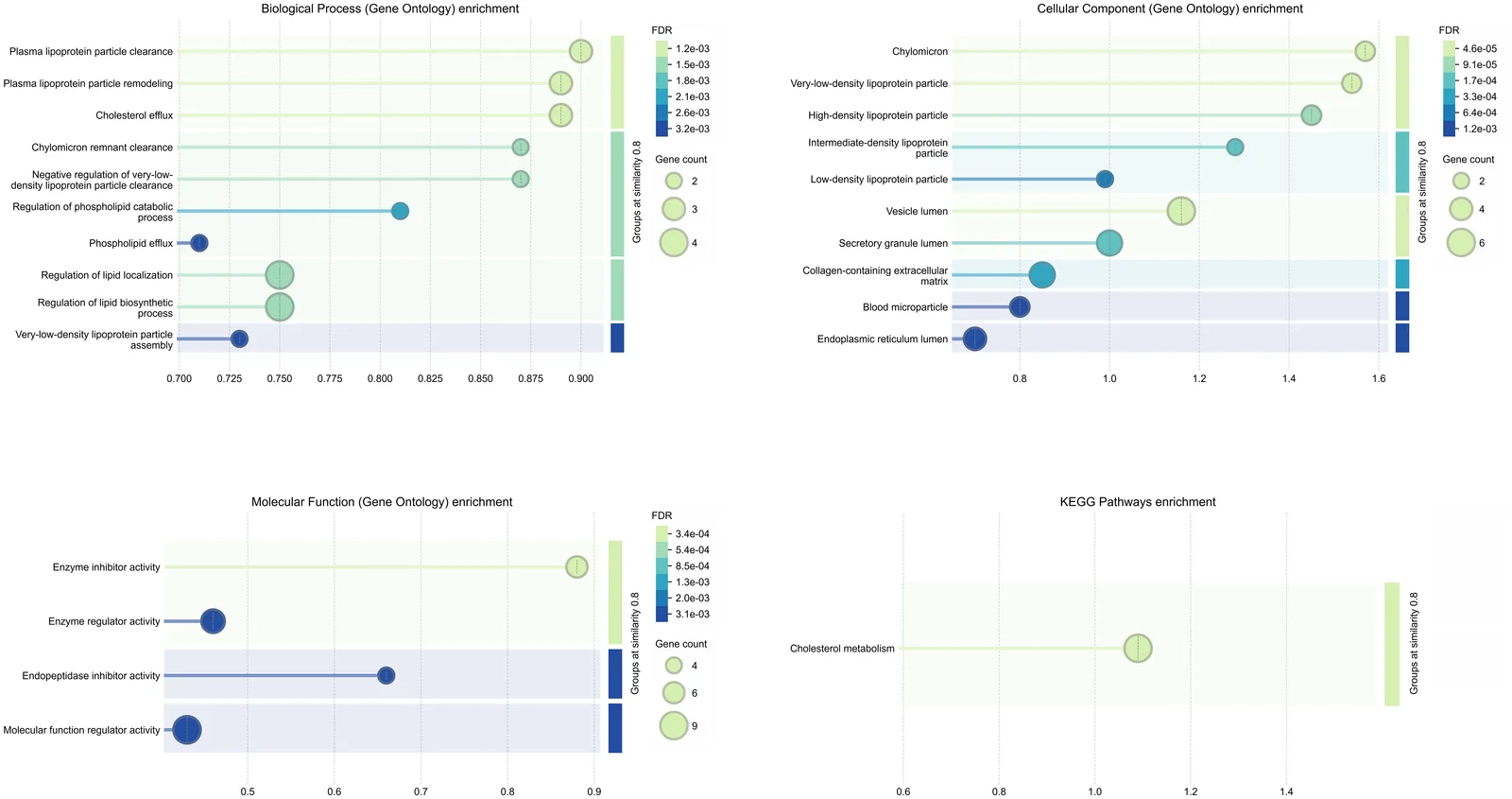
Gene Ontology (GO) analysis of the differentially expressed proteins was performed, including biological process (BP), molecular function (MF), and cellular component (CC), along with KEGG pathway analysis.

### Prognostic association analysis in the proteomic cohort

The differentially expressed proteins were further analyzed in the Proteome-Phenome Atlas database for prognostic association. The results showed that APOC1 was the only protein whose hazard ratio (HR) had a 95% confidence interval that did not cross 1, indicating a statistically significant association with prognosis. These findings suggest that APOC1 may be closely associated with the prognosis of patients with ACLF. Therefore, APOC1 was selected as the key candidate protein for further investigation (**Figure 5**).

**Figure 5:**
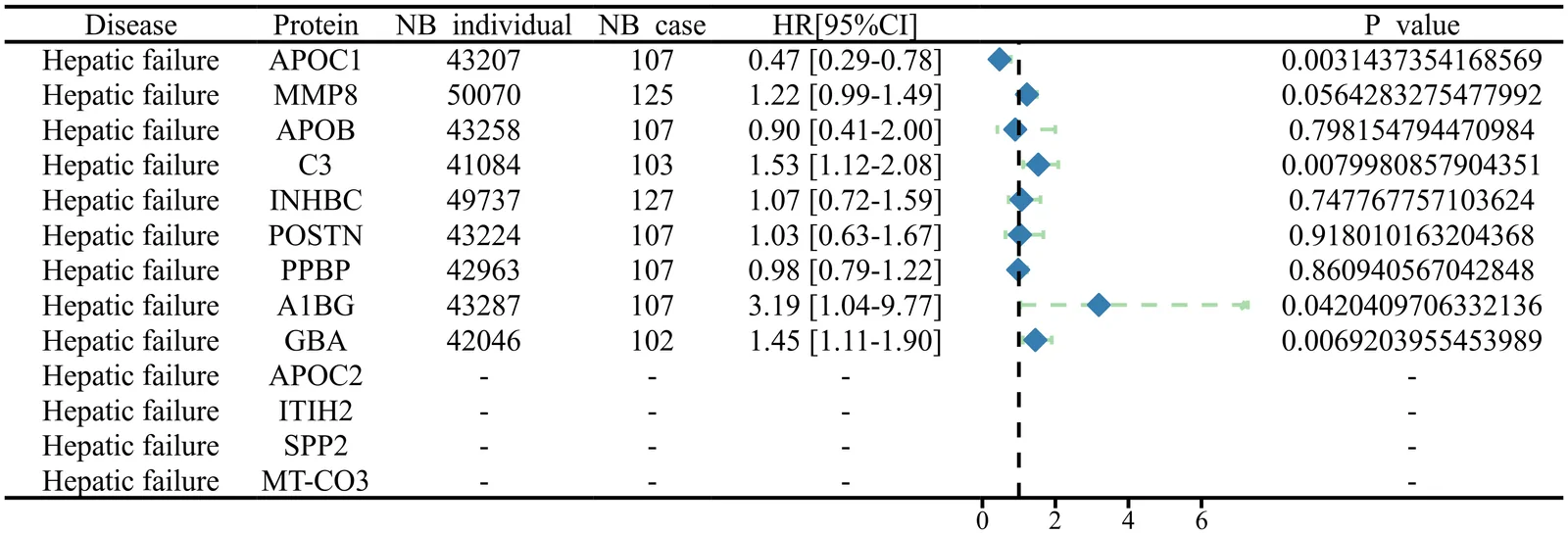
Forest plot of the proteomic study.

### ELISA verification and survival analysis

A total of 74 participants were included in the validation cohort, comprising 47 patients in the survival group (64%) and 27 in the non-survival group (36%). ELISA validation revealed that the median serum APOC1 level in the survival group was 9.26 μg/mL (interquartile range [IQR], 6.88–13.93), which was significantly higher than that in the non-survival group (5.79 μg/mL; IQR, 4.73–7.54; P < 0.001), suggesting a potential protective role. Further subgroup analysis according to disease stage demonstrated that APOC1 levels progressively decreased with disease progression, with highly significant differences among stages (P < 0.001): levels were highest in the early-stage group at 11.51 μg/mL (IQR, 7.77–14.73), decreased to 7.51 μg/mL (IQR, 6.17–9.56) in the mid-stage group, and reached the lowest level in the advanced-stage group at 4.83 μg/mL (IQR, 3.99–5.28). Kaplan–Meier survival analysis further confirmed the prognostic value of APOC1, showing that patients in the high-expression group had a significantly higher cumulative survival rate compared with the low-expression group (log-rank P = 0.001). These findings indicate that high APOC1 expression is significantly associated with improved survival outcomes and may serve as an effective biomarker for prognostic risk stratification in patients with ACLF (**Figure 6**, **Tables 2**, **3**).

**Figure 6:**
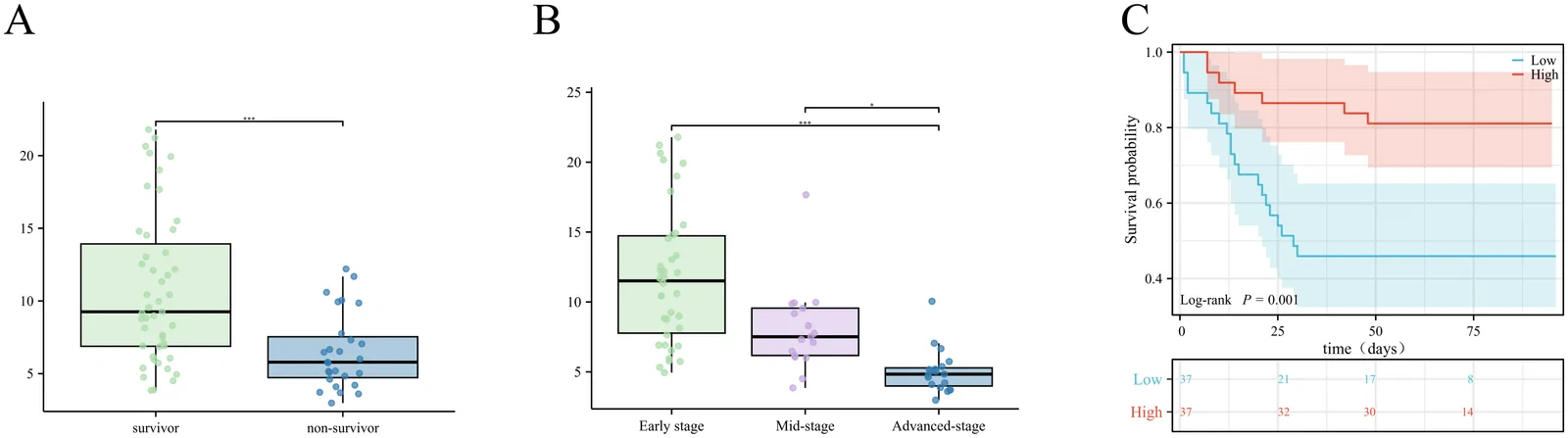
**(A)** ELISA analysis of APOC1 in patients with ACLF. Comparison of serum APOC1 concentrations between the survival group and the non-survival group. **(B)** Based on APOC1 protein concentration, patients with acute-on-chronic liver failure (ACLF) were classified into early-, intermediate-, and advanced-stage groups. **(C)** Kaplan–Meier survival analysis according to APOC1 protein levels.

**Table 2:** Analysis of serum APOC1 levels detected by ELISA between survivor and non-survivor groups in patients with ACLF.

Variable	Survivor N = 47 (64%)^1^	Non-survivor N = 27 (36%)^1^	p-value^2^
APOC1	9.26 [6.88, 13.93]	5.79 [4.73, 7.54]	<0.001

1 Median [IQR].

2 Wilcoxon rank sum test.

**Table 3:** Based on APOC1 protein concentration, patients with acute-on-chronic liver failure (ACLF) were classified into early-, intermediate-, and advanced-stage groups.

Variable	Earlystage N = 38 (51%)^1^	Mid-stage N = 17 (23%)^1^	Advanced stage N = 19 (26%)^1^	p-value^2^
APOC1	11.51 [7.77, 14.73]	7.51 [6.17, 9.56]	4.83 [3.99, 5.28]	<0.001
clinicaloutcome				<0.001
survivor	33 (86.84%)	11 (64.71%)	3 (15.79%)	
non-survivor	5 (13.16%)	6 (35.29%)	16 (84.21%)	

1 Median [IQR]; n (%).

2 Kruskal-Wallis rank sum test; Fisher’s Exact Test for Count Data with simulated p-value (based on 2000 replicates).

### Logistic regression and comparison with conventional scores

Univariable logistic regression analysis showed that several indicators, including APOC1, hemoglobin, platelet count, total bilirubin, albumin, INR, MELD, and COSSH-ACLF II, were significantly associated with the prognosis of patients with ACLF (all P < 0.05). Subsequently, variables with P < 0.1 in the univariable analysis were subsequently entered into the multivariable logistic regression model. An exploratory prognostic model incorporating four predictors was constructed based on the regression coefficients derived from the multivariable analysis. To explore the potential incremental prognostic value of APOC1, APOC1 was separately combined with conventional scoring systems, including MELD, MELD-Na, and COSSH-ACLF II, to construct combined risk prediction models. In the present study, these combined models were successfully constructed and showed numerically greater prognostic discrimination than the corresponding conventional scoring systems alone. However, given the limited number of outcome events and the absence of internal validation and independent external validation, these findings should be regarded as exploratory.


Model 1 = 2.49544007 − 0.2750463 × APOC1 − 0.0163505 × PLT + 0.00672926 × TBIL.

The results indicated that lower APOC1, lower platelet count, higher total bilirubin were associated with an increased risk of poor prognosis.


Model 2 =−0.650033−0.2368772×APOC1 + 0.06585839×MELD.

The results suggested that lower APOC1 levels and higher MELD scores were associated with a higher risk of poor prognosis.


Model 3=−1.4894659−0.2418647×APOC1 + 0.09205198×MELD−Na.

The results suggested that lower APOC1 levels and higher MELD-Na scores were associated with a higher risk of poor prognosis.


Model 4=−6.0323973−0.2286713×APOC1 + 0.97574408×COSSH−II.

The results suggested that lower APOC1 levels and higher COSSH-II scores were associated with a higher risk of poor prognosis.

The predicted probability of an individual experiencing a poor prognosis was calculated using the following formula: $P=\frac{1}{1+e-Logit(P)}$, where P represents the probability of the outcome event. In this study, poor prognosis was defined as death within 90 days, and the dependent variable was coded as follows: poor prognosis = 1 and survival = 0.

The results showed that APOC1 could be incorporated separately into conventional prognostic scoring systems, including MELD, MELD-Na, and COSSH-ACLF II, to construct combined risk prediction models. Within the present cohort, these combined models demonstrated numerically higher apparent discrimination than the corresponding conventional scoring systems alone, suggesting that APOC1 may provide complementary prognostic information. ROC curve analysis showed that serum APOC1 had an apparent area under the curve (AUC) of 0.764 for predicting an unfavorable outcome, with an optimal cutoff value of 6.7591 μg/mL. In the present cohort, serum APOC1 levels below this threshold were associated with a higher risk of an unfavorable outcome. Compared with total bilirubin, the international normalized ratio (INR), MELD, and COSSH-ACLF II, the APOC1-based combined models yielded numerically higher apparent AUC values in this dataset. However, because differences in AUC between the models were not adequately validated, these findings reflect only numerical differences observed in the current sample and should not be interpreted as evidence of a definitive statistical advantage of the combined models (**Tables 4**–**6**; **Figure 7**).

**Table 4:** Multivariate logistic regression analysis.

Characteristics	Total(N)	Univariate analysis		Multivariate analysis	
		Odds ratio (95% CI)	P value	Odds ratio (95% CI)	P value
APOC1(ug/ml)	74	0.747 (0.626 – 0.892)	0.001	0.760 (0.613 – 0.941)	0.012
lymphocytes (10^9^/L)	74	0.951 (0.426 – 2.124)	0.902		
Monocytes (10^9^/L)	74	2.171 (0.315 – 14.974)	0.432		
Neutrophils (10^9^/L)	74	1.093 (0.901 – 1.326)	0.367		
HGB(g/L)	74	0.978 (0.953 – 1.004)	0.091	1.014 (0.973 – 1.057)	0.507
PLT (10^9^/L)	74	0.984 (0.972 – 0.996)	0.007	0.984 (0.968 – 1.000)	0.045
ALT(U/L)	74	1.000 (0.999 – 1.000)	0.222		
AST(U/L)	74	1.000 (0.999 – 1.000)	0.281		
TBIL(μmol/L)	74	1.005 (1.001 – 1.009)	0.009	1.007 (1.001 – 1.013)	0.022
ALB(g/L)	74	0.857 (0.762 – 0.965)	0.011	0.875 (0.728 – 1.051)	0.152
CREA(μmol/L)	74	1.010 (0.991 – 1.030)	0.293		
INR	74	3.148 (1.474 – 6.725)	0.003	1.665 (0.804 – 3.446)	0.170
K(mmol/L)	74	0.668 (0.266 – 1.681)	0.392		
Na(mmol/L)	74	1.027 (0.920 – 1.146)	0.633		
Cl(mmol/L)	74	1.029 (0.942 – 1.122)	0.528		
PCT	74	1.222 (0.912 – 1.639)	0.180		

**Table 5:** Logistic regression analysis.

Model	Variables included	Prediction equation
Model 1	APOC1, PLT, TBIL,	Logit(P) = 2.49544007 - 0.2750463 × APOC1 - 0.0163505 × PLT + 0.00672926 × TBIL
Model 2	APOC1, MELD	Logit(P) = -0.650033 - 0.2368772 × APOC1 + 0.06585839 × MELD
Model 3	APOC1, MELD-Na	Logit(P) = -1.4894659 - 0.2418647 × APOC1 + 0.09205198 × MELD-Na
Model 4	APOC1, COSSH-II	Logit(P) = -6.0323973 - 0.2286713 × APOC1 + 0.97574408 × COSSH-II

**Table 6:** Diagnostic performance of serum APOC1 for predicting 90-day poor prognosis in patients with ACLF.

Predictive variable	Cut-off value	Sensitivity	Specificity	Accuracy	TP	TN	FP	FN	PPV	NPV	Youden index
APOC1 (μg/mL)	6.7591	0.66667	0.76596	0.72973	18	36	11	9	0.62069	0.80000	0.43262

TP, true positive; TN, true negative; FP, false positive; FN, false negative; PPV, positive predictive value; NPV, negative predictive value.

The cut-off value, sensitivity, specificity, accuracy, true positive (TP), true negative (TN), false positive (FP), false negative (FN), positive predictive value (PPV), negative predictive value (NPV), and Youden index of serum APOC1 were calculated for predicting 90-day poor prognosis.

**Figure 7:**
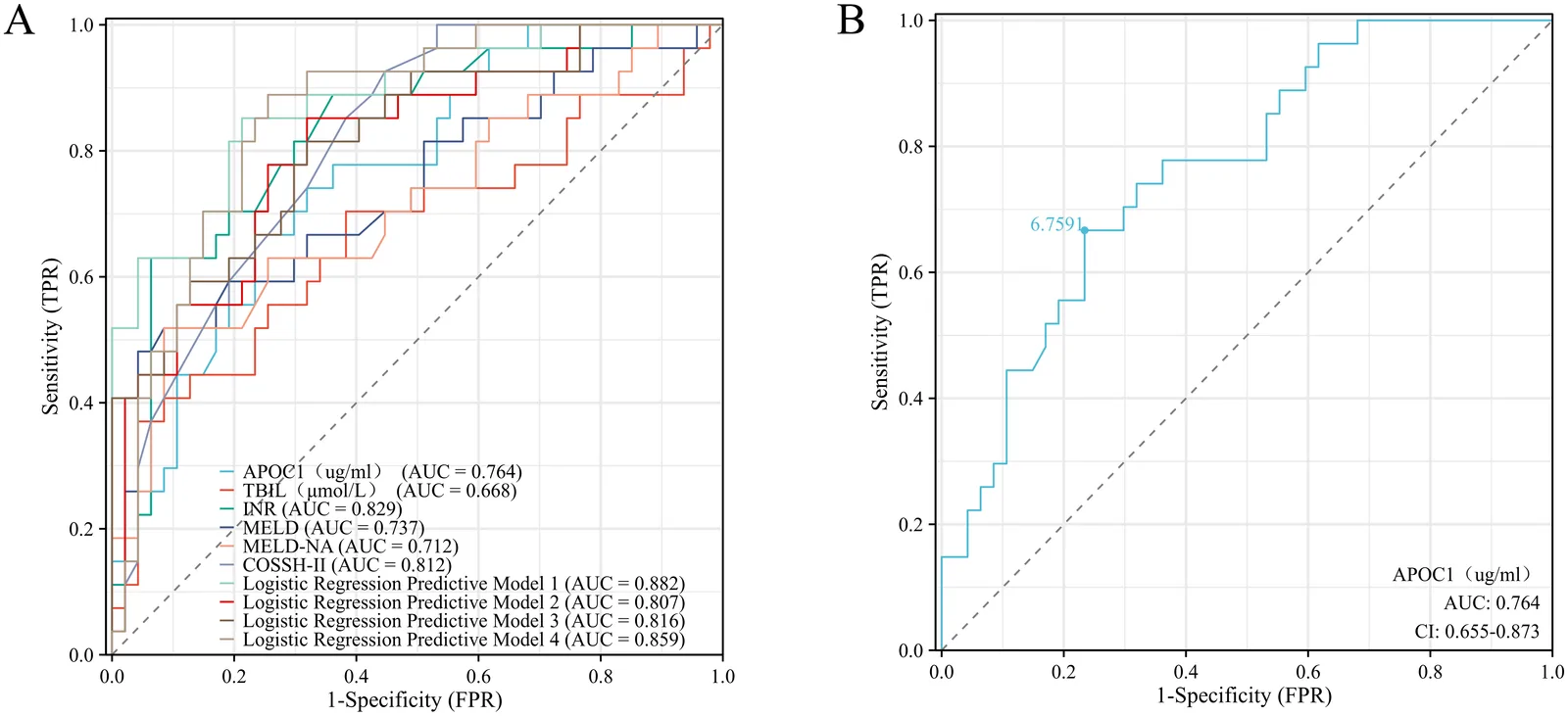
**(A)** ROC curve analysis was conducted to compare the predictive performance of APOC1, the logistic regression model, INR, TBIL, MELD score, MELD-Na score, and COSSH-II score. **(B)** ROC curve analysis of serum APOC1 concentration for prognosis prediction, with an AUC of 0.764 and an optimal cut-off value of 6.7591.

### Single-cell RNA sequencing

Single-cell analysis of 14 liver tissue specimens from three healthy controls and three patients with cirrhosis showed that APOC1 was predominantly expressed in macrophages, providing a basis for subsequent cell-based experiments (**Figure 8**).

**Figure 8:**
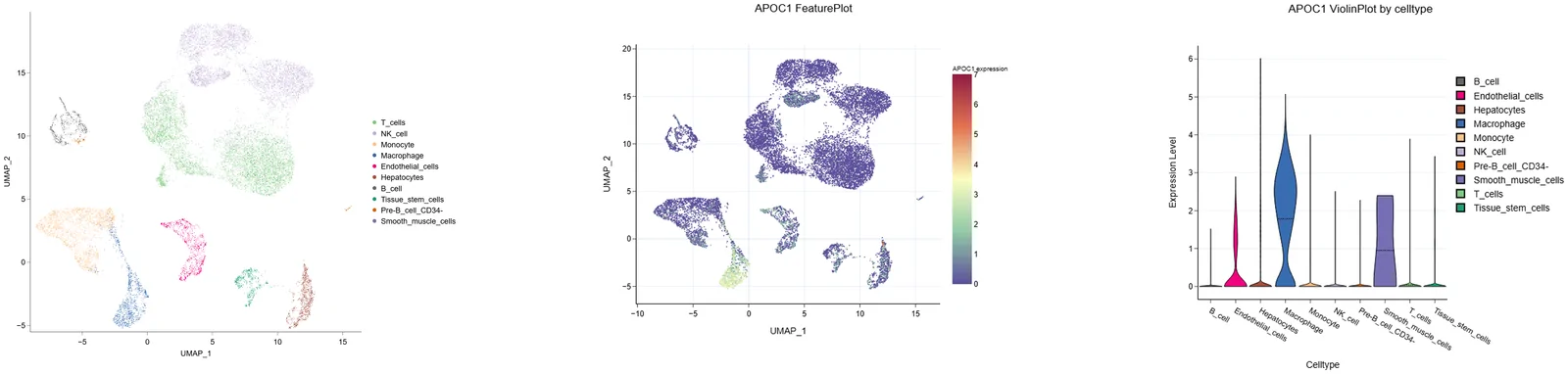
Single-cell localization analysis of APOC1 in liver tissue. Following dimensionality reduction using UMAP, liver tissue cells were clustered and analyzed by single-cell RNA sequencing (10×). Different colors indicate different cell types, including macrophages (brown), hepatocytes (red), and T cells (green). APOC1 expression was found to be predominantly localized in macrophages.

### Molecular docking of APOC1 with saikosaponin D and trans-resveratrol

Molecular docking was performed using AutoDock Vina to evaluate the potential interactions of saikosaponin D and trans-resveratrol with APOC1. The docking poses were visualized using Discovery Studio 2019 and PyMOL 2.6. The predicted binding energies of saikosaponin D and trans-resveratrol with APOC1 were −7.8 and −6.7 kcal/mol, respectively. These results suggest that both compounds may form energetically favorable interactions with APOC1 under the computational docking conditions. However, as these findings were derived solely from in silico analysis, their binding affinity and functional relevance require further validation through biochemical, cell-based, and *in vivo* experiments (**Figure 9**).

**Figure 9:**
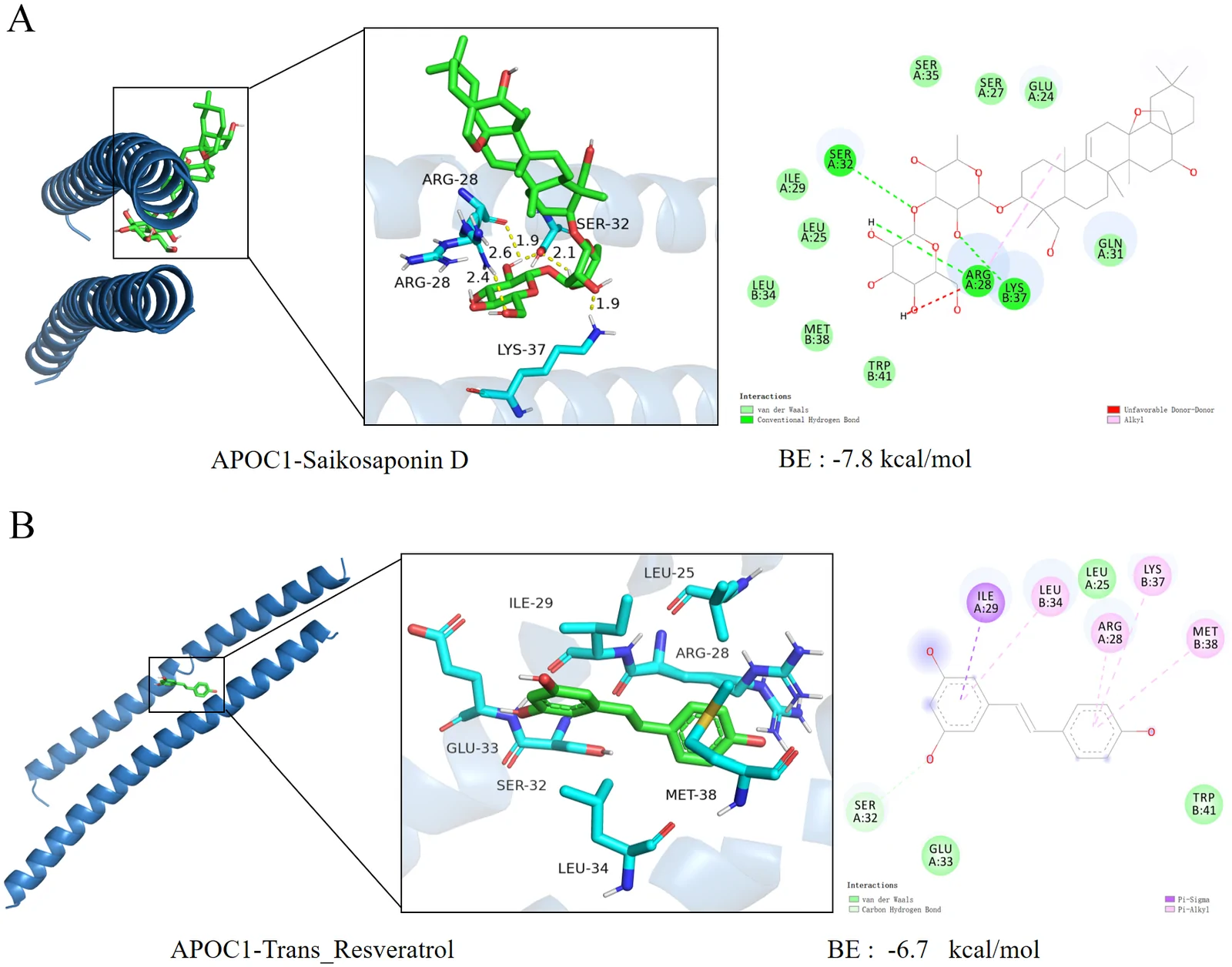
**(A)** Saikosaponin D exhibited a binding energy of −7.8 kcal/mol. **(B)** Resveratrol exhibited a binding energy of −6.7 kcal/mol.

## Discussion

ACLF is a syndrome of acute hepatic decompensation occurring in the setting of chronic liver disease and is characterized by abrupt onset, rapid progression, high short-term mortality, and a high risk of multiple organ dysfunction (3, 4, 26, 27). Increasing evidence suggests that ACLF is not merely a consequence of hepatocellular necrosis, but rather the result of multiple interacting processes, including severe liver injury, dysregulated systemic inflammation, immune dysfunction, and metabolic reprogramming (4, 5, 28–34). Given the marked clinical heterogeneity of ACLF, conventional laboratory parameters and existing scoring systems still have limitations in early risk stratification and individualized prognostic assessment. Therefore, the identification of novel prognostic biomarkers is of considerable importance for improving outcome prediction and further elucidating the underlying mechanisms of ACLF (11, 12, 35, 36).

In the present study, proteomic analysis was first performed to compare patients in the survival and non-survival groups with ACLF, leading to the identification of APOC1 as a differentially expressed protein that was significantly upregulated in the survival group. This finding was further validated by ELISA, which consistently showed higher APOC1 levels in the survival group than in the non-survival group, supporting the consistency of the proteomic results. In addition, ROC curve analysis demonstrated that APOC1 had good discriminative ability for predicting the prognosis of patients with ACLF, with an AUC of 0.764, which was higher than that of conventional indicators such as total bilirubin and the MELD score. Further regression analysis showed that APOC1 was a protective factor for prognosis in ACLF, with an odds ratio (OR) of 0.747 (95% CI, 0.598-0.935; P = 0.011). Together, these findings preliminarily suggest that APOC1 may be associated with the prognosis of patients with ACLF and may provide complementary prognostic information beyond conventional scoring systems. However, the present study included only 74 patients and 27 death events, and the prognostic model incorporated four predictors, raising concerns regarding model overfitting and instability of parameter estimates. Moreover, the absence of independent external validation may have resulted in optimistic estimates of the observed AUC and model performance. Accordingly, the current model should be regarded as exploratory and is not yet suitable for clinical decision-making or generalization to other patient populations. The incremental predictive value, stability, and generalizability of APOC1-based models require further confirmation through rigorous internal and external validation in larger, independent, multicenter cohorts with sufficient outcome events. Therefore, APOC1 should currently be considered a candidate prognostic biomarker rather than a clinically established prognostic tool for ACLF (16, 21, 37).

APOC1 is a member of the apolipoprotein C family and is mainly synthesized and secreted by the liver, where it plays an important role in lipid transport and lipoprotein metabolism. As the central organ of lipid metabolism, the liver is critical for maintaining cholesterol homeostasis, lipoprotein synthesis, and energy balance (38). Patients with ACLF are typically in a state of marked hypercatabolism and negative energy balance, and dysregulated lipid metabolism is recognized as an important pathological feature of disease progression (18, 39). In the setting of severe liver injury, impaired hepatocellular synthetic function is often accompanied by abnormal levels of multiple apolipoproteins. Therefore, the higher expression of APOC1 observed in the survival group in this study may suggest that elevated APOC1 reflects a relative preservation of hepatocellular synthetic capacity and lipid metabolic regulation, indicating that these patients retain a certain degree of metabolic reserve despite acute insult. The presence of such metabolic reserve may help maintain internal homeostasis, attenuate disease progression, and thereby improve clinical outcomes.

Notably, the biological role of APOC1 is not limited to lipid metabolism. Recent studies have shown that APOC1 is also involved in the regulation of inflammatory responses, immune cell function, and host defense, particularly in relation to the functional state of the monocyte-macrophage system (34, 40). One of the central pathological features of ACLF is the coexistence of excessive systemic inflammatory activation and immune dysregulation. The amplified inflammatory cascade not only directly aggravates hepatocellular injury, but also substantially affects short-term prognosis through the induction of extrahepatic organ dysfunction (41–44). Therefore, the higher APOC1 expression observed in the survival group, together with its identification as a protective factor, suggests that APOC1 may not only reflect better hepatic synthetic function and metabolic status, but may also participate in the regulation of inflammatory responses and the maintenance of immune homeostasis. In other words, higher APOC1 levels may help buffer the secondary damage caused by uncontrolled inflammation, thereby contributing to improved survival outcomes in patients with ACLF. Nevertheless, this interpretation is currently based mainly on clinical association analyses, and the specific molecular mechanisms require further clarification through cell-based and animal studies.

The ROC analysis in the present study showed that APOC1 had better prognostic performance than several commonly used clinical indicators, a finding with clear clinical relevance. At present, prognostic assessment in ACLF relies largely on MELD score, Child-Pugh score, and laboratory indicators such as serum bilirubin, coagulation parameters, and creatinine. However, these indices mainly reflect the severity of organ injury or disease status and provide relatively limited information regarding metabolic reserve and alterations in inflammatory-immune networks. In contrast, APOC1, as a serum protein, offers several practical advantages, including ease of sampling, established detection methods, and good reproducibility. If its predictive value is further confirmed in larger, multicenter cohorts, APOC1 may serve as an important complementary biomarker to existing scoring systems for early risk stratification, dynamic disease monitoring, and prognostic evaluation in ACLF, thereby providing additional support for precision clinical management (32, 35, 36, 45).

In addition, the present findings suggest that differences in prognosis among patients with ACLF may be closely related to the complex interplay among metabolism, inflammation, and immunity (39). Previous ACLF research has focused mainly on inflammatory cytokine storms, hepatocellular necrosis, and organ failure, whereas comparatively less attention has been paid to the role of lipid metabolism-related proteins in disease evolution (46). The present study showed that high APOC1 expression was associated with survival benefit and functioned as a protective factor, providing further support for the concept that ACLF is, at least in part, an immunometabolic disorder. APOC1 may not simply represent an accompanying biomarker change, but rather a potential intersection point linking abnormal lipid metabolism, inflammatory amplification, and stress adaptation. This finding not only provides a novel candidate biomarker for prognostic evaluation in ACLF, but also offers a new perspective for exploring disease intervention targets from the standpoint of metabolic regulation.

Several limitations of this study should be acknowledged. First, this was a single-center retrospective study with a relatively small sample size. Only 74 patients with acute-on-chronic liver failure (ACLF) were included, among whom 27 experienced the endpoint of death. Therefore, the study may be subject to selection bias, and the generalizability of the findings requires further validation in larger, multicenter, prospective cohorts. Second, the prognostic model has statistical limitations. Model development and performance assessment were conducted using the same cohort, without validation in an independent external cohort. Consequently, the estimated predictive performance may be subject to optimism bias. Owing to the retrospective nature of clinical sample collection and the limited sample size, internal validation using bootstrap resampling or similar methods was not performed. Therefore, the stability, calibration, and clinical generalizability of the model require further evaluation. Third, although the ELISA validation results were consistent with the proteomic findings, and ROC curve and regression analyses suggested that APOC1 had a certain degree of prognostic discrimination and was associated with clinical outcomes in patients with ACLF, the observational nature of this study precludes the establishment of a causal relationship between APOC1 levels and improved survival. Moreover, APOC1 levels may be influenced by multiple factors, including the etiology of the underlying liver disease, infection status, nutritional status, baseline lipid metabolism, and treatment interventions. Therefore, residual confounding cannot be completely excluded. Finally, the primary aim of this study was to identify potential prognostic biomarkers for ACLF and to evaluate the clinical value of APOC1 in prognostic assessment. Based on the present findings and previously published evidence, we hypothesize that APOC1 may exert protective effects by modulating macrophage function, inflammatory cytokine secretion, immune homeostasis, and hepatocyte survival. However, the present study did not include macrophage functional assays, hepatocyte co-culture experiments, or *in vivo* animal models, nor did it directly investigate the cellular origin, associated signaling pathways, or functional regulatory mechanisms of APOC1. Accordingly, the proposed mechanisms remain hypothetical and are based on clinical associations and existing literature. Further *in vitro* and *in vivo* functional studies, together with validation in large-scale clinical cohorts, are required to clarify the biological functions, molecular mechanisms, and translational potential of APOC1 in ACLF.

In conclusion, proteomic analysis identified a higher expression trend of APOC1 in the survival group of patients with ACLF, which was further supported by consistent findings from ELISA validation. ROC curve analysis showed that APOC1 demonstrated a certain degree of apparent prognostic discrimination in the present cohort, while regression analysis suggested that APOC1 levels were associated with prognosis in patients with ACLF. These findings suggest that APOC1 may serve as a promising biomarker for prognostic assessment in ACLF. Its potential role may be related to the maintenance of lipid metabolic homeostasis, preservation of hepatic synthetic reserve, and participation in inflammatory and immune regulation. The present study not only provides a novel candidate biomarker for prognostic evaluation in ACLF, but also offers new evidence for understanding the mechanisms underlying ACLF progression from an immunometabolic perspective. Further large-scale clinical validation and mechanistic studies are needed to clarify the biological functions and clinical utility of APOC1 in ACLF.

## Data Availability

The data presented in the study are deposited in the China National Gene Bank (CNGBdb, https://db.cngb.org/) repository, accession number: CNP0004833.
